# Establishment of a genetic transformation system for cordycipitoid fungus *Cordyceps chanhua*

**DOI:** 10.3389/fmicb.2024.1333793

**Published:** 2024-06-27

**Authors:** Ruihang Cai, Yu Xiao, Jiajia Xing, Kongjian Yu, Xiaola Li, Yiqiu Chai

**Affiliations:** ^1^Zhejiang Institute of Subtropical Crops, Zhejiang Academy of Agricultural Sciences, Wenzhou, China; ^2^State Key Laboratory of Rice Biology and Ministry of Agriculture Key Laboratory of Molecular Biology of Crop Pathogens and Insects, Institute of Biotechnology, Zhejiang University, Hangzhou, China; ^3^College of Environmental and Resource Sciences, Zhejiang A&F University, Hangzhou, China

**Keywords:** *Cordyceps chanhua*, medicinal fungus, protoplast preparation, protoplast regeneration, genetic transformation

## Abstract

*Cordyceps chanhua* is a well-known edible and medicinal mushroom with a long history of use in China, and it contains a variety of secondary metabolites with interesting bioactive ingredients. However, recent researches have mainly focused on cultivation conditions, secondary metabolite compositions and pharmacological activities of *C. chanhua*, the lack of an efficient and stable genetic transformation system has largely limited further research on the relationship between secondary metabolites and biosynthetic gene clusters in *C. chanhua*. In this study, single-factor experiments were used to compare the effects of different osmotic stabilizers, enzyme concentrations and enzyme digestion times on protoplast yield, and we found that the highest yield of 5.53 × 10^8^ protoplasts/mL was obtained with 0.7 M mannitol, 6 mg/mL snail enzyme and 4 h of enzyme digestion time, and the regeneration rate of protoplasts was up to approximately 30% using 0.7 M mannitol as an osmotic stabilizer. On this basis, a PEG-mediated genetic transformation system of *C. chanhua* was successfully established for the first time, which lays the foundation for further genetic transformation of *C. chanhua*.

## 1 Introduction

*Cordyceps chanhua*, also known as *Cordyceps cicada*, is an entomopathogenic fungus of the genus *Cordyceps* in the family Claviciptaceae that can infest the nymphs of insects (e.g., *Cicada flammata*, *Crytotympana pustulata*) and form fruiting bodies on their body surfaces (Li et al., 2020, 2021). The fruiting bodies of *C. chanhua* have been used as traditional Chinese medicine for more than 1,600 years (Li et al., 2019). The wild-type strain is mainly distributed in eastern and southwestern China. Moreover, as an edible and medicinal resource, *C. chanhua* has high research and development value due to its various bioactive components, such as adenosine, amino acids, fatty acids, polysaccharides and ergosterol (Zhao et al., 2014; Sun et al., 2018; Zhang et al., 2019; Nxumalo et al., 2020; Raethong et al., 2023). Modern pharmacological studies have also confirmed its pharmacological efficacy as renoprotective, anti-inflammatory, antitumor, antidiabetic and immunomodulatory effects (Taofiq et al., 2016; He et al., 2018; Xu et al., 2018; Zhang et al., 2018; Zheng et al., 2018; Wang et al., 2019). Biomolecular research is crucial for the exploitation of these bioactive metabolites. However, at genetic level, the functional pharmacological studies of *C. chanhua* has been hindered by the lack of an efficient genetic tools such as transformation systems.

The development of genetic transformation techniques is a precondition that allows the targeting and efficiently modifying genes and may reveal the function of target genes (Li et al., 2017). Currently, there exist several common techniques for genetically transforming filamentous fungi, such as protoplast-mediated transformation (PMT), Agrobacterium-mediated transformation (AMT), shock-wave-mediated transformation (SWMT), electroporation (EP), biolistic transformation/particle bombardment (BT) and some genome editing tools [e.g., zinc-finger nucleases (ZFNs), transcription activator-like effector nucleases (TALENs), and the clustered regularly interspaced short palindromic repeat/associated protein 9 (CRISPR/Cas9)] (Díza et al., 2019; Ullah et al., 2020; Liang et al., 2021; Zhang et al., 2022). Overall, these manipulation techniques above mainly include random DNA integration, gene-targeting technology, RNA technology, and modern gene-editing technologies (Ullah et al., 2020). Despite these, fungal cell wall components are highly variable among different strains, no universal genetic transformation method can be applied for all filamentous fungal strains. Hence, it is of utmost importance to establish species-specific genetic manipulation methods for further research at the molecular level.

Specifically, the PMT fungal genetic manipulation method has the advantages of high transformation efficiency, gentle reaction conditions, and low cost (Díza et al., 2019). This approach involves the use of commercially accessible hydrolyzing enzymes to eliminate fungal cell walls, resulting in the production of protoplasts. Following this, certain chemical agents, such as polyethylene glycol (PEG) or calcium ions, facilitate the fusion and uptake of exogenous nucleic acids and protoplasts (Su et al., 2021). In similar applications, the selection of an appropriate cell wall degrading enzyme and its concentration is vital (Agrawal et al., 2015, 2022). Since the first successful transformation of yeast protoplasts using the PMT method by Hinnen et al. (1978), the method has become increasingly widely used and has been established in a number of filamentous fungi (e.g., *Pleurotus eryngii*, *Ganoderma lucidum*, *Cordyceps militaris* and *Candida glycerinogenes*) (Hinnen et al., 1978; Zhang et al., 2016; Lou et al., 2019; You et al., 2021; Zhang et al., 2022). It is noteworthy that the transformation rate is highly dependent on the cell wall-degrading enzymes (Zhang et al., 2016, 2022). Even, the source of variability for the osmotic stabilizers should be well considered as they are pivotal in upholding osmotic pressure and averting protoplast deformation/collapse (Dobrowolska and Staczek, 2009; Díza et al., 2019; Su et al., 2021). Up to date, an optimized PMT approach for fungi particularly in the case of *C. chanhua* is still controversial. More efforts should be developed about the protoplast yield as affected by the enzyme digestion time and concentrations.

In this study, we explored the optimization of protoplast preparation of *C. chanhua* strain 022017-3 and successfully established a simple, efficient and stable PEG-mediated protoplast transformation as a novel tool for the genetic transformation system of *C. chanhua*. In addition, PEG-mediated homologous recombination was used to test the transformation system of strain 022017-3 protoplasts. This is the first research report on the successful establishment of a genetic transformation system for *C. chanhua*, which is of great significance for the subsequent analyses of functional genes and the regulatory mechanisms of effective medicinal components.

## 2 Materials and methods

### 2.1 Fungal strains and culture conditions

The wild-type *C. chanhua*, strain 022017-3 was reserved in the Zhejiang Institute of Subtropical Crops, Zhejiang Academy of Agricultural Sciences, Wenzhou, China, and used to create the genetic system in this study. The mycelium of strain 022017-3 was picked and maintained on potato dextrose agar (PDA) medium at 24°C for 6–7 days. One 2 × 2 cm mycelial block on PDA solid medium was cut with a sterilized knife, placed in a 1.5 mL sterile centrifuge tube, fully ground with a sterile grinding rod, inoculated into 50 mL of potato dextrose broth (PDB) medium containing a concentration of 100 μg/mL ampicillin, and cultured at 24°C with 150 rpm for 3–4 days to collect mycelium.

### 2.2 Resistance gene selection and fungal sensitivity test

The hygromycin B is widely used as an antibiotic screening agent in fungal genetic transformation (Gritz and Davies, 1983; Ning et al., 2022; Li et al., 2023). The antibiotic sensitivity of *C. chanhua* strain 022017-3 was determined in PDA medium with different concentrations of hygromycin B (200, 250, 300, 350, 400, 450 μg/mL). The control group did not receive antibiotics. Petri dishes were incubated for 5 days at 24°C.

### 2.3 Preparation of protoplasts

#### 2.3.1 Mycelium collection

The liquid medium was centrifuged at 5,000 rpm for 10 min to obtain the mycelium and then washed thrice with 30 mL of 0.7 M osmotic stabilizer.

#### 2.3.2 Fungal cell wall lysis

Snail enzyme was used as a solvent, and 0.7 mol/L osmotic stabilizer was added to prepare the final concentration of snail enzyme solution. Then, 10 mL of enzyme solution was added to 5 g of mycelium and incubated for a period of time at 37°C and 150 rpm.

#### 2.3.3 Protoplast collection

The above enzymatic solution was filtered with four layers of sterilized lens paper, and the filtrate was collected into a sterile 50 mL centrifuge tube at 4°C and 5,000 rpm for 10 min. The precipitate was the desired protoplasts.

#### 2.3.4 Protoplast washing

Precooled STC buffer at 4°C was added to the protoplasts, and the volume was fixed to 20 mL and centrifuged at 5,000 rpm for 10 min at 4°C. This procedure was repeated twice. Protoplasts were observed under 40× microscopy, and the final concentration was adjusted to 10^8^ protoplasts/mL by STC solution.

### 2.4 Single factor experiments of protoplast preparation

On the basis of pre-experiments, to select an efficient method for protoplast preparation, different osmotic stabilizers (0.7 M NaCl, 0.7 M KCl, 0.7 M MgSO4, 0.7 M mannitol and 0.7 M sucrose), snailase concentrations (2, 4, 6, 8, 10 and 12 mg/mL) and enzyme digestion times (2.5, 3, 3.5, 4, 4.5 and 5 h) were selected as influencing factors by single factor experiments with protoplast yield as the evaluation index.

### 2.5 Single factor experiments of protoplast regeneration

The regeneration of protoplasts was affected by the type of osmotic stabilizers in regeneration medium. In this experiment, four commonly used osmotic stabilizers (0.7 M KCl, 0.7 M NaCl, 0.7 M mannitol and 0.7 M sucrose) were selected, and a one-way test was carried out with osmotic stabilizer type as the influencing factor and the regeneration rate of protoplasts as the evaluation index. The medium composition used in this study was listed in Table 1. The concentration of protoplasts was adjusted from 10^8^ protoplasts/mL to 10^3^ protoplasts/mL with STC solution, and then 200 μL was taken and poured into melted 200 mL of regeneration solid medium and shaken slowly to mix well. PDA medium without osmotic stabilizers was used as a control. All the above plates were incubated at 24°C in the dark for 6 days. The protoplast regeneration rate = (the number of colonies in experimental group − the number of colonies in control group)/the number of protoplasts * 100%.

**Table 1:** Medium composition for *Cordyceps chanhua* genetic transformation.

Medium	Composition
Potato dextrose agar (PDA)	Potato 200 g/L, dextrose 20 g/L, agar 15 g/L
Potato dextrose broth (PDB)	Potato 200 g/L, dextrose 20 g/L
Regeneration solid medium	Potato 200 g/L, dextrose 20 g/L, osmotic stabilizer 0.7 M, agar 15 g/L
Regeneration liquid medium	Potato 200 g/L, dextrose 20 g/L, osmotic stabilizer 0.7 M
Antibiotic medium	Potato 200 g/L, dextrose 20 g/L, agar 15 g/L, 300 μg/mL hygromycin B

### 2.6 Plasmid construction

To construct the deletion cassette, 1–1.5 kb fragments upstream (*Up*) and downstream (*Down*) of the target gene and the hygromycin phosphotransferase gene (*hyg*) need to be obtained. The plasmid pKOV21 was used to obtain the *hyg* gene in this study. Fragments 1–1.5 kb upstream and downstream were amplified from the genomic DNA of strain 022017-3 using the indicated primers (Table 2). The double-joint PCR method was carried out to construct the combined knockout cassette (Yu et al., 2004). The above three fragments were purified with an Agarose Gel Extraction Kit PCR Clean-up Kit (VWI Biotech) and ligated by double-joint PCR in the order of “*Up-hyg-Down*.” The “*Up-hyg-Down*” fragment was assembled into the pGEM-T easy vector by the pGEM-T easy Vector System, Promega.

**Table 2:** PCR primers for deletion cassette construction and transformants validation.

Primers	Oligonucleotide sequence (5′–3′)	Uses	Length of amplified sequence
up-F	AGGTAGTCCTTATATAGGGATAGC	Up flanks’ amplification for deletion cassete	1.5 kb
up-R	GCTCCTTCAATATCATCTTCTCTCGAGCAGAGGGAAGCGACAACT		
down-F	AGAGTAGATGCCGACCGAACAAGAAAGATGGAGGAAGCAATGTCG	Down flanks’ amplification for deletion cassete	1.5 kb
down-R	CGGAAGAACGCAGTGTATCG		
hyg-F	CGAGAGAAGATGATATTGAAGGAGC	*hyg* gene amplification and diagnostic PCR for transformants	1.4 kb
hyg-R	TCTTGTTCGGTCGGCATCTACTCTA		

### 2.7 PEG-mediated protoplast transformation and verification of transformants

The 12 μg deletion cassettes were added to 850 μL of 10^8^ mL^−1^ protoplasts and placed on ice for 25 min. Then, 1 mL of PTC solution was slowly added to the mixture and incubated on ice for 25 min. Subsequently, the above mixture was added to 20 mL of regeneration liquid medium and incubated overnight at 24°C and 100 rpm to revive the protoplasts. The revived protoplasts were added to 150 mL of prewarmed regeneration solid medium and gently mixed, after which the mixture was poured into a petri dish to form a thin layer and cultivated in a dark environment at 24°C for 1 to 2 days. Antibiotic medium containing 300 μg/mL hygromycin B was poured over the above regeneration solid medium petri dishes for screening in the dark at 24°C, and transformants were observed for approximately 5–7 days.

The transformants were picked out three times in antibiotic medium, and the third generation was incubated in PDB for genomic DNA extraction and amplified by PCR using the indicated primers to verify the accuracy of the transformants (Table 2).

### 2.8 Statistical analysis

Each experiment was performed at least in triplicate. Data were presented as the mean ± standard deviation (SD). The data analysis was performed using Microsoft Excel and SPSS software (SPSS Inc., Ver.19, IL, United States). A one-way analysis of variance (ANOVA) was used to analyze the significance difference using Duncan’s multiple range test (*p* ≤ 0.05).

## 3 Results

### 3.1 Resistance gene selection and fungal sensitivity test

**Figure 1:**
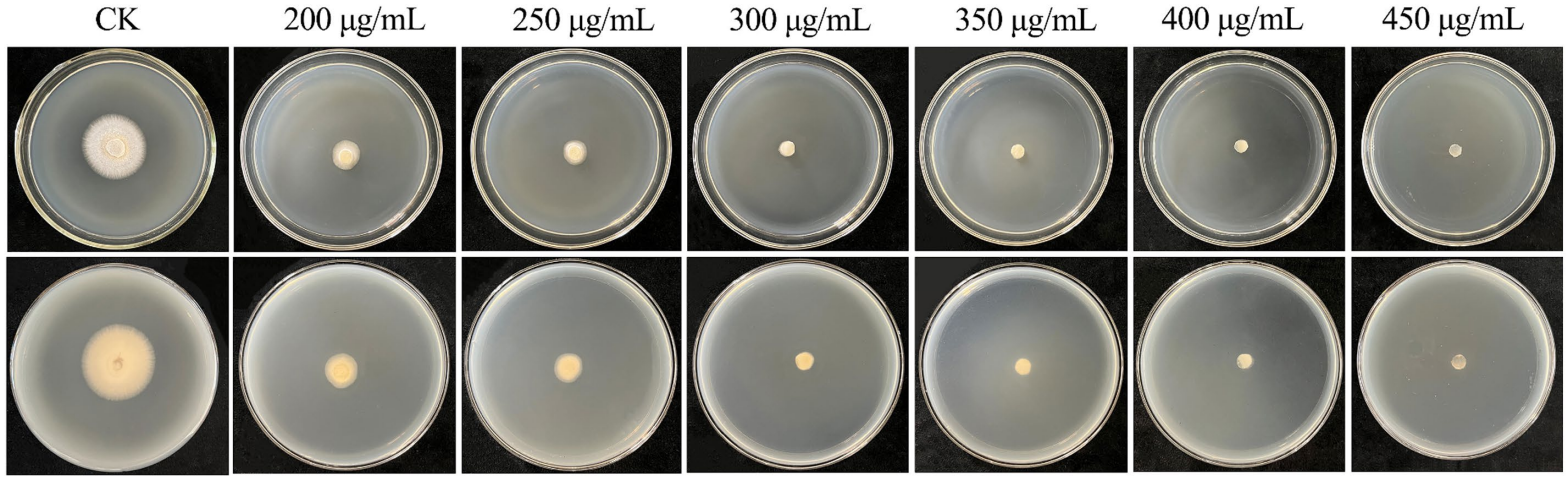
Sensitivity of wild type strain 022017-3 toward hygromycin B. PDA medium was supplemented with different concentrations of hygromycin B (200–450 μg/mL) and PDA without hygromycin B (CK) were incubated for 5 days at 24°C.

Suitable selection markers are important for the establishment of genetic transformation systems in filamentous fungi, which can be used to distinguish transformants from nontransformants (Ruiz-Díez, 2002). In this study, the sensitivity of *C. chanhua* to hygromycin B was tested, and it was found that the growth of strain 022017-3 was inhibited at a concentration of 300 μg/mL (Figure 1).

### 3.2 Effect of different osmotic stabilizers on protoplast yield

**Figure 2:**
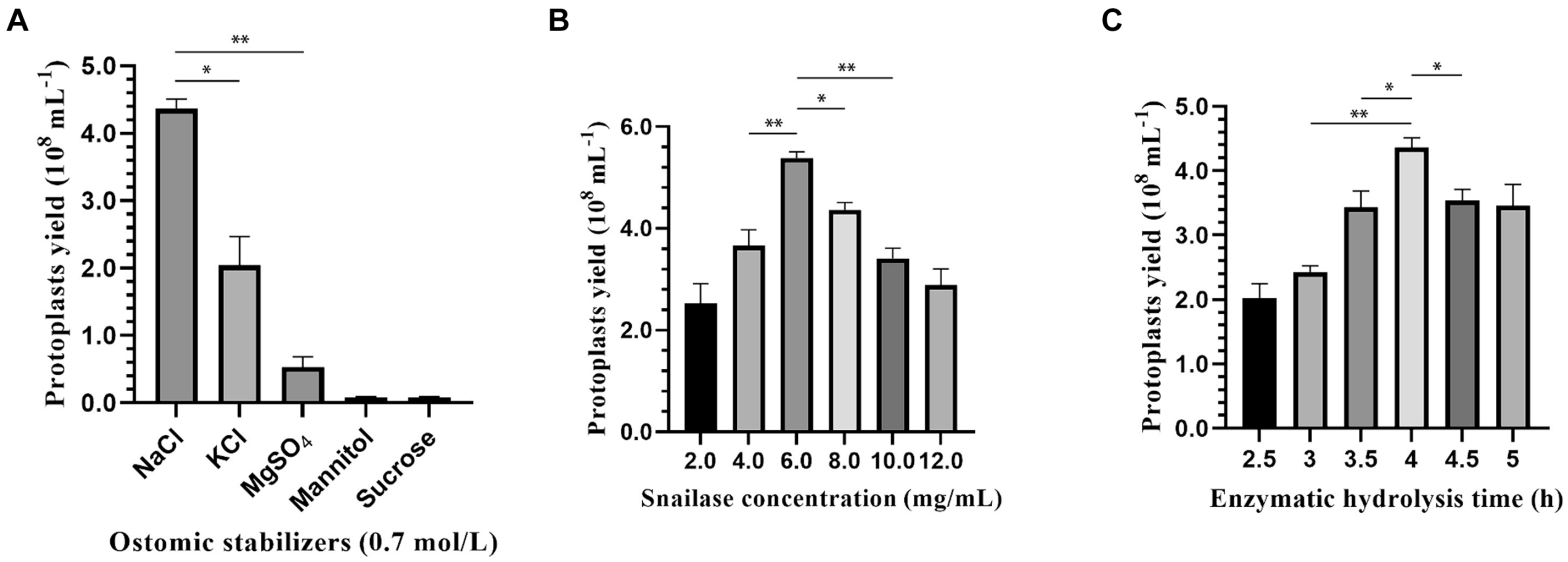
Effect of osmotic stabilizers, enzymatic hydrolysis concentration and enzymatic hydrolysis time on protoplasts preparation of strain 022017-3. **(A)** Osmotic stabilizers. **(B)** Snailase concentration. **(C)** Enzymatic hydrolysis time (^**^p < 0.01 and ^*^p < 0.05).

An optimal osmotic stabilizer can maintain cell conformation and prevent cell membrane rupture. As shown in Figure 2A, 0.7 M NaCl was the most effective osmotic stabilizer for protoplast preparation, with the highest protoplast yield of 4.36 × 10^8^ protoplasts/mL, followed by 0.7 M KCl at 2.13 × 10^8^ protoplasts/mL. In addition, with mannitol and sucrose as the osmotic stabilizer, the mycelium would be suspended in the upper layer of the liquid, and it was difficult to precipitate it by increasing the centrifugation speed and prolonging the centrifugation time, which would reduce the amount of mycelium and decrease the accuracy of the results.

### 3.3 Effect of enzymatic hydrolysis concentration on protoplast yield

There was a significant difference in the number of protoplasts cleaved by different concentrations of snailase. Figure 2B shows that with the increase in enzymatic hydrolysis concentration from 2 to 6 mg/mL, the protoplasts had a rapid growth rate. At an enzymatic hydrolysis concentration of 6 mg/mL, the protoplast yield reached 5.53 × 10^8^ protoplasts/mL. As the enzymatic hydrolysis concentration was further elevated, there was a notable deceleration in the growth rate of protoplasts.

### 3.4 Effect of enzymatic hydrolysis time on protoplast yield

The enzymatic hydrolysis time also had a great influence on the production of protoplasts. Figure 2C shows that the protoplast yield increased as the enzymatic hydrolysis time increased from 2.5 to 4 h and slowly decreased after 4 h. In the preparation of protoplasts, 4 h was the most suitable enzymatic hydrolysis time, and the highest protoplast yield reached 4.36 × 10^8^ protoplasts/mL.

### 3.5 Regeneration of *Cordyceps chanhua* strain 022017-3 protoplasts

**Figure 3:**
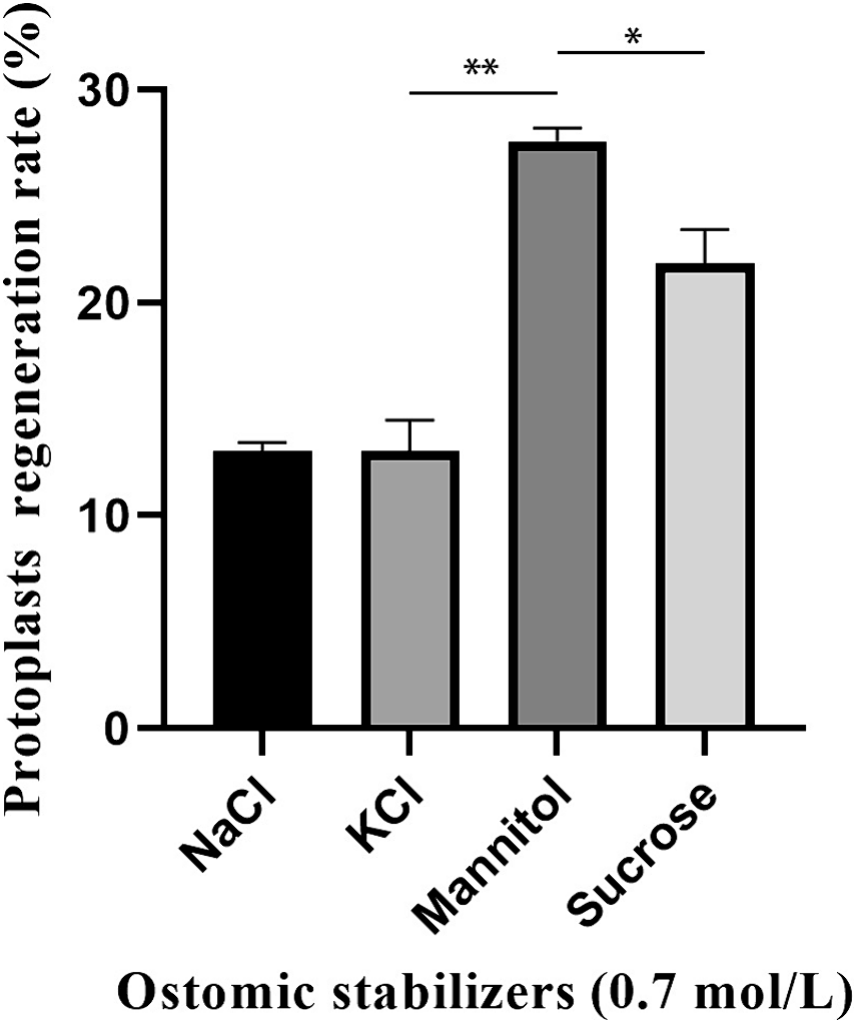
Effect of osmotic stabilizers on protoplasts regeneration of strain 022017-3 (^**^p < 0.01 and ^*^p < 0.05).

Four osmotic stabilizers (0.7 M KCl, 0.7 M NaCl, 0.7 M mannitol and 0.7 M sucrose) were used to determine the regeneration rate of *C. chanhua* protoplasts. It was found that 0.7 M mannitol had the best effect, and the regeneration rate reached nearly 30% (Figure 3).

### 3.6 Verification of transformants during protoplast transformation

**Figure 4:**
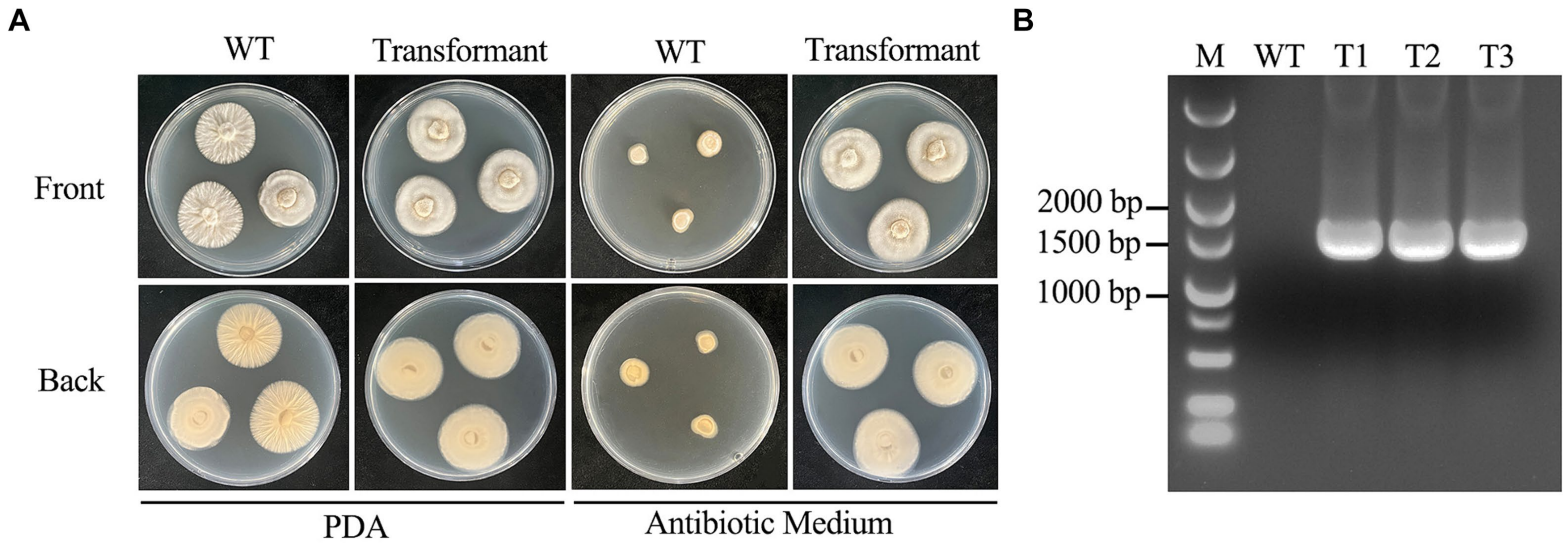
Morphological comparison of wild type strain 022017-3 with transformants and confirmation of transformants. **(A)** Phenotypes of wild type strain 022017-3 and transformant on PDA and antibiotic medium at 24°C for 6 days. **(B)** Diagnostic PCR to identify the transformants (M, Marker; WT, Wild Type; T1, Transformant 1; T2, Transformant 2; T3, Transformant 3).

In this study, we validated the transformation system by transferring the *hyg* gene into the *C. chanhua* genome. The strain successfully transferred with the *hyg* gene could grow normally in antibiotic medium. Eleven single colonies grew in antibiotic medium, those colonies were picked out three times in antibiotic medium, and the third generation was verified by diagnostic PCR. Finally, three correct transformants were obtained (Figure 4).

## 4 Discussion

*C. chanhua* is a significant traditional Chinese medicine with a diverse array of bioactive constituents, indicating potential for broader applications through the investigation of these metabolites. Nevertheless, the lack of a genetic transformation platform has notably constrained the exploration of functional genes and secondary metabolite synthesis mechanisms in *C. chanhua*. While genetic transformation systems have been successfully developed for *C. militaris* within the *Cordyceps* genus, the transformation protocols vary considerably across different species (Sun et al., 2017; Meng et al., 2022). Hence, it is imperative to thoroughly examine the transformation manipulations in *C. chanhua*.

The yield and regeneration quality of protoplasts are influenced by multiple factors, including osmotic stabilizers, enzymatic hydrolysis concentration and time (Díza et al., 2019). Among them, osmotic stabilizers mainly play a role in maintaining the osmotic pressure of the cytoplasmic membrane system. Cell wall-degrading enzymes may damage the membrane system, inactivate the released protoplasts and make regeneration difficult, so an optimal osmotic stabilizer is needed to protect the protoplasts so that the internal and external pressure of the protoplasts remains balanced. To illustrate, 0.6–1.2 M sorbitol solution is used in the protoplast preparation of *Amphichorda guana* (Liang et al., 2021), *Neurospora crassa* (Case et al., 1979) and *Aspergillus oryzae* (Hahm and Batt, 1988) to maintain osmotic stability in protoplasts. Additionally, there exists a clear correlation between the concentration of hydrolyzing enzymes and the resulting protoplast yield (Wu et al., 2018). Increasing the concentration of hydrolyzing enzymes within a specific range enhances the liberation of protoplasts. However, once a certain threshold is reached, further increases in concentration do not lead to a significant rise in the number of protoplasts released. Based on the principle of cost-effectiveness, it is necessary to specify the optimal enzymatic concentration when the maximum protoplast yield is reached. Currently, the commonly used cell wall-degrading enzymes include snailase, driselase and lywallzyme (Han et al., 2020; Ning et al., 2022; Xie et al., 2022). Snailase is a complex enzyme that consists of over 20 different types of enzymes, e.g., cellulase, pectinase, mannanase, glucoamylase, chitinase and lipase (Liang et al., 2012). In present study, snailase was selected to degrade the cell wall of *C. chanhua* due to its low cost and is one of the effective enzymes for the preparation of fungal protoplasts. Furthermore, enzymatic hydrolysis time also has a considerable effect on protoplast yield. The young mycelium will be cleaved alongside the release of protoplasts, followed by the relatively older mycelium. Consequently, inadequate enzymatic hydrolysis duration may result in incomplete degradation of cell walls, while excessive duration may lead to potential damage to protoplasts, both of which will result in a lower protoplast yield and affect the regeneration rate of protoplasts (Agrawal et al., 2015; Ning et al., 2022).

In addition, transformation experiments showed that *hyg* gene could be successfully transferred into the genome of *C. chanhua* 022017-3 which ensured the mitotic stability of the transformants. About 11 transformants could be obtained from 12 μg of the deletion cassettes, and 3 of them were positive as identified by diagnostic PCR. The desirable transformation efficiencies proved that the established genetic transformation system could satisfy the needs for further genetic manipulation and research.

In fungi, the availability of transformation systems is crucial for investigating the functional roles of genes associated with the synthesis of specific metabolites or enzymes. Resistance genes are significant contributors to the genetic transformation of fungi, and mutant strains carrying resistance genes can be screened from wild-type strains by resistance screening medium. The hygromycin phosphotransferase gene is widely used as an antibiotic transformation marker in fungi, including *Eutypella* sp. (Ning et al., 2022), *Poria cocos* (Xie et al., 2022) and *Gloeophyllum trabeum* (Li et al., 2023). Here, we determined that the lowest hygromycin B inhibition concentration in *C. chanhua* 022017–3 was 300 μg/mL.

## 5 Conclusion

This study represented the first successful example of PMT system of the cordycipitoid fungus *C. chanhua* strain 022017-3 with excellence in transformation efficiencies. Preparation and regeneration of protoplasts are the basis for genetic transformation. We observed that the highest yield of 5.53 × 10^8^ protoplasts/mL was obtained with 0.7 M mannitol, 6 mg/mL snail enzyme and 4 h of enzyme digestion time, and the regeneration rate of protoplasts was up to approximately 30% using 0.7 M mannitol as an osmotic stabilizer. However, the PMT system also has some limitations, such as low transformation efficiency and regeneration rate in *C. chanhua*, which require further studies to improve. In conclusion, the genetic transformation of *C. chanhua* described in this work will provide access to further investigate the functional genes and secondary metabolite synthesis mechanisms in *C. chanhua*.

## Data availability statement

The original contributions presented in the study are publicly available. The Whole Genome Shotgun project has been deposited at DDBJ/ENA/GenBank under the accession JBELXZ000000000.

## Author contributions

RC: Data curation, Formal analysis, Funding acquisition, Investigation, Methodology, Software, Writing – original draft, Writing – review & editing. YX: Formal analysis, Methodology, Writing – review & editing. JX: Data curation, Formal analysis, Writing – review & editing. KY: Data curation, Writing – review & editing. XL: Validation, Writing – review & editing. YC: Funding acquisition, Project administration, Writing – review & editing.

## Supplementary material

SUPPLEMENTARY FIGURE S1 Construction of deletion cassette by PCR. **(A)** *Up* and *down* fragments of target gene. **(B)** *Hyg* fragment (M, Marker).

SUPPLEMENTARY FIGURE S2 *Up*, *hyg* and *down* fragments of deletion cassette were assembled by double-joint PCR (M, Marker).

## References

[ref1] Agrawal R. Bhadana B. Chauhan P. S. Adsul M. Kumar R. Gupta R. P.. (2022). Understanding the effects of low enzyme dosage and high solid loading on the enzyme inhibition and strategies to improve hydrolysis yields of pilot scale pretreated rice straw. Fuel 327:125114. doi: 10.1016/j.fuel.2022.125114

[ref2] Agrawal R. Satlewal A. Gaur R. Mathur A. Kumar R. Gupta R. P.. (2015). Pilot scale pretreatment of wheat straw and comparative evaluation of commercial enzyme preparations for biomass saccharification and fermentation. Biochem. Eng. J. 102, 54–61. doi: 10.1016/j.bej.2015.02.018

[ref3] Case M. E. Schweizer M. Sidney R. K. Giles N. H. (1979). Efficient transformation of *Neurospora crassa* by utilizing hybrid plasmid DNA. Proc. Natl. Acad. Sci. U.S.A. 76, 5259–5263. doi: 10.1073/pnas.76.10.5259, PMID: 159454 PMC413120

[ref4] Díza A. Villanueva P. Oliva V. Gil-Durán C. Fierro F. Chávez R.. (2019). Genetic transformation of the filamentous fungus *Pseudogymnoascus verrucosus* of Antarctic origin. Front. Microbiol. 10:2675. doi: 10.3389/fmicb.2019.02675, PMID: 31824460 PMC6883257

[ref5] Dobrowolska A. Staczek P. (2009). Development of transformation system for *Trichophyton rubrum* by electroporation of germinated conidia. Curr. Genet. 55, 537–542. doi: 10.1007/s00294-009-0264-8, PMID: 19629488

[ref6] Gritz L. Davies J. (1983). Plasmid encoded hygromycin B resistance: the sequence of hygromycin B phosphotransferase gene and its expression in *Escherichia Coli* and *Saccharomyces Cerevisiae*. Gene 25, 179–188. doi: 10.1016/0378-1119(83)90223-8, PMID: 6319235

[ref7] Hahm Y. T. Batt C. A. (1988). Genetic transformation of an *argB* mutant of *Aspergillus oryzae*. Appl. Environ. Microbiol. 54, 1610–1611. doi: 10.1128/aem.54.6.1610-1611.1988, PMID: 16347669 PMC202705

[ref8] Han G. Y. Liu N. Li C. L. Tu J. Li Z. Sheng C. Q. (2020). Discovery of novel fungal lanosterol 14#-demethylase (CYP51)/histone deacetylase (HDAC) dual inhibitors to treat azole-resistant candidiasis. J. Med. Chem. 63, 5341–5359. doi: 10.1021/acs.jmedchem.0c00102, PMID: 32347094

[ref9] He L. F. Shi W. J. Liu X. C. Zhao X. H. Zhang Z. C. (2018). Anticancer action and mechanism of ergosterol peroxide from *Paecilomyces cicadae* fermentation broth. Int. J. Mol. Sci. 19:3935. doi: 10.3390/ijms19123935, PMID: 30544579 PMC6321734

[ref10] Hinnen A. Hicks J. B. Fink G. R. (1978). Transformation of yeast. Proc. Natl. Acad. Sci. U.S.A. 75, 1929–1933. doi: 10.1073/pnas.75.4.1929, PMID: 347451 PMC392455

[ref11] Li W. R. Ayers C. Huang W. P. Schilling J. S. Gullen D. Zhang J. W. (2023). A laccase gene reporting system that enables genetic manipulations in a brown rot wood decomposer fungus *Gloeophyllum trabeum*. Microbiol. Spectr. 11, e04246–e04222. doi: 10.1128/spectrum.04246-22, PMID: 36651769 PMC9927100

[ref12] Li Z. Z. Hywel-Jones N. L. Luan F. G. Zhang S. L. Sun C. S. Chen Z. A.. (2020). Biodiversity of cordycipitoid fungi associated with *Isaria cicadae* I: literature study. Mycosystema 39, 2191–2201,

[ref13] Li I. C. Lin S. Tsai Y. T. Hsu J. H. Chen Y. L. Lin W. H.. (2019). *Cordyceps cicadae* mycelia and its active compound HEA exert beneficial effects on blood glucose in type 2 diabetic db/db mice. J. Sci. Food Agric. 99, 606–612. doi: 10.1002/jsfa.9221, PMID: 29952113

[ref14] Li Z. Z. Luan F. G. Hywel-Jones N. L. Zhang S. L. Chen M. J. Huang B.. (2021). Biodiversity of cordycipitoid fungi associated with *Isaria cicadae* Miquel II: teleomorph discovery and nomenclature of chanhua, an important medicinal fungus in China. Mycosystema 40, 95–107,

[ref15] Li D. D. Tang Y. Lin J. Cai W. W. (2017). Methods for genetic transformation of filamentous fungi. Microb. Cell Factories 16:168. doi: 10.1186/s12934-017-0785-7, PMID: 28974205 PMC5627406

[ref16] Liang M. Li W. Qi L. D. Chen G. C. Cai L. Yin W. B. (2021). Establishment of a genetic transformation system in guanophilic fungus *Amphichorda guana*. *J. Fung*i 7:138. doi: 10.3390/jof7020138, PMID: 33672933 PMC7918455

[ref17] Liang K. H. Zhang Q. H. Cong W. (2012). Enzyme-assisted aqueous extraction of lipid from microalgae. J. Agric. Food Chem. 60, 11771–11776. doi: 10.1021/jf302836v, PMID: 23072503

[ref18] Lou H. W. Ye Z. W. Yu Y. H. Lin J. F. Guo L. Q. Chen B. X.. (2019). The efficient genetic transformation of *Cordyceps militaris* by using mononuclear protoplasts. Sci. Hortic. 243, 307–313. doi: 10.1016/j.scienta.2018.08.043

[ref19] Meng G. L. Wang X. P. Liu M. Q. Wang F. Liu Q. Z. Dong C. H. (2022). Efficient CRISPR/Cas9 system based on autonomously replicating plasmid with an AMA1 sequence and precisely targeted gene deletion in the edible fungus, *Cordyceps militaris*. Microb. Biotechnol. 15, 2594–2606. doi: 10.1111/1751-7915.14107, PMID: 35829671 PMC9518986

[ref20] Ning Y. D. Hu B. Yu H. B. Liu X. Y. Jiao B. H. Lu X. L. (2022). Optimization of protoplast preparation and establishment of genetic transformation system of an arctic-derived fungus *Eutypella* sp. Front. Microbiol. 13:769008. doi: 10.3389/fmicb.2022.769008, PMID: 35464961 PMC9019751

[ref21] Nxumalo W. Elateeq A. A. Sun Y. F. (2020). Can *Cordyceps cicadae* be used as an alternative to *Cordyceps militaris* and *Cordyceps sinensi*s?—A review. J. Ethnopharmacol. 257:112879. doi: 10.1016/j.jep.2020.112879, PMID: 32305637

[ref22] Raethong N. Thananusak R. Cheawchanlertfa P. Prabhakaran P. Rattanaporn K. Laoteng K.. (2023). Functional genomics and systems biology of *Cordyceps* species for biotechnological applications. Curr. Opin. Biotechnol. 81:102939. doi: 10.1016/j.copbio.2023.102939, PMID: 37075529

[ref23] Ruiz-Díez R. (2002). Strategies for the transformation of filamentous fungi. J. Appl. Microbiol. 92, 189–195. doi: 10.1046/j.1365-2672.2002.01516.x 11849345

[ref24] Su Z. Z. Dai M. D. Zhu J. N. Zeng Y. L. Lu X. J. Liu X. H.. (2021). An efficient genetic manipulation protocol for dark septate endophyte *Falciphora oryzae*. Biotechnol. Lett. 43, 2045–2052. doi: 10.1007/s10529-021-03171-5, PMID: 34390483

[ref25] Sun Y. F. Sum Y. Wang Z. A. Han R. L. Lu H. F. Zhang J. L.. (2018). *Isaria cicadae* conidia possess antiproliferative and inducing apoptosis properties in gynaecological carcinoma cells. Mycoloy 8, 327–334. doi: 10.1080/21501203.2017.1386243, PMID: 30123653 PMC6059127

[ref26] Sun D. Zhang M. Xie C. R. Guo X. W. Xu H. H. Gao H. T.. (2017). Establishment of genetic transformation system of *Cordyceps militaris* using PEG mediated method. Chin. Biotechnol. 34, 76–82,

[ref27] Taofiq O. Martins A. Barreiro M. F. Ferreira I. C. F. R. (2016). Anti-inflammatory potential of mushroom extracts and isolated metabolites. Trends Food Sci. Technol. 50, 193–210. doi: 10.1016/j.tifs.2016.02.005

[ref28] Ullah M. Xia L. Xie S. Sun S. (2020). CRISPR/Cas9-based genome engineering: a new breakthrough in the genetic manipulation of filamentous fungi. Biotechnol. Appl. Biochem. 67, 835–851. doi: 10.1002/bab.2077, PMID: 33179815

[ref29] Wang Y. B. He P. F. He L. Huang Q. R. Cheng J. W. Li W. Q.. (2019). Structural elucidation, antioxidant and immunomodulatory activities of a novel heteropolysaccharide from cultured *Paecilomyces cicadae* (Miguel.) Samson. Carbohydr. Polym. 216, 270–281. doi: 10.1016/j.carbpol.2019.03.104, PMID: 31047067

[ref30] Wu C. H. Jiang P. Zhao J. Fu H. H. (2018). High efficiency of protoplast preparation for artificially cultured *Ulva prolifera* (Ulvophyceae, Chlorophyta). J. Oceanol. Limnol. 36, 1806–1811. doi: 10.1007/s00343-018-7058-0

[ref31] Xie Z. N. Zhong C. Liu X. L. Wang Z. L. Zhou R. R. Xie J.. (2022). Genome editing in the edible fungus *Poria cocos* using CRISPR-Cas9 system integrating genome-wide off-target prediction and detection. Front. Microbiol. 13:966231. doi: 10.3389/fmicb.2022.966231, PMID: 36071963 PMC9441760

[ref32] Xu Z. C. Yan X. T. Song Z. Y. Li W. Zhao W. B. Ma H. H.. (2018). Two heteropolysaccharides from *Isaria cicadae* Miquel differ in composition and potentially immunomodulatory activity. Int. J. Biol. Macromol. 117, 610–616. doi: 10.1016/j.ijbiomac.2018.05.164, PMID: 29802926

[ref33] You H. Sun B. Li N. Xu J. W. (2021). Efficient expression of heterologous genes by the introduction of the endogenous glyceraldehyde-3-phosphate dehydrogenase gene intron 1 in *Ganoderma lucidum*. Microb. Cell Factories 20:164. doi: 10.1186/s12934-021-01654-8, PMID: 34419069 PMC8379801

[ref34] Yu J. H. Hamari Z. Han K. H. Seo J. A. Reyes-Dominguez Y. Scazzocchio C. (2004). Double-joint PCR: a PCR-based molecular tool for gene manipulations in filamentous fungi. Fungal Genet. Biol. 41, 973–981. doi: 10.1016/j.fgb.2004.08.001, PMID: 15465386

[ref35] Zhang X. F. Hu Q. B. Weng Q. F. (2019). Secondary metabolites (SMs) of *Isaria cicadae* and *Isaria tenuipes*. RSC Adv. 9, 172–184. doi: 10.1039/c8ra09039d, PMID: 35521576 PMC9059538

[ref36] Zhang Q. P. Olatunji O. J. Chen H. X. Tola A. J. Oluwaniyi O. O. (2018). Evaluation of the anti-diabetic activity of polysaccharide from *Cordyceps cicadae* in experimental diabetic rats. Chem. Biodivers. 15:e1800219. doi: 10.1002/cbdv.201800219, PMID: 29874416

[ref37] Zhang Q. Zhao L. T. Shen M. Y. Liu J. Y. Li Y. R. Xu S.. (2022). Establishment of an efficient polyethylene glycol (PEG)-mediated transformation system in *Pleurotus eryngii* var. *ferulae* using comprehensive optimization and multiple endogenous promoters. J. Fungi 8:186. doi: 10.3390/jof8020186, PMID: 35205941 PMC8876744

[ref38] Zhang C. Zong H. Zhuge B. Lu X. Y. Fang H. Y. Zhu J. L.. (2016). Protoplast preparation and polyethylene glycol (PEG)-mediated transformation of *Candida glycerinogenes*. Biotechnol. Bioprocess Eng. 21, 95–102. doi: 10.1007/s12257-015-0686-8

[ref39] Zhao J. Xie J. Wang L. Y. Li S. P. (2014). Advanced development in chemical analysis of *Cordyceps*. J. Pharm. Biomed. Anal. 87, 271–289. doi: 10.1016/j.jpba.2013.04.025, PMID: 23688494

[ref40] Zheng R. Zhu R. Li X. L. Li X. Y. Shen L. L. Chen Y.. (2018). N6-(2-hydroxyethyl) adenosine from *Cordyceps cicadae* ameliorates renal interstitial fibrosis and prevents inflammation via TGF-β1/Smad and NF-κB B signaling pathway. Front. Physiol. 9:1229. doi: 10.3389/fphys.2018.01229, PMID: 30233405 PMC6131671

