# Supplementary figures and images for: Establishment of a genetic transformation system for cordycipitoid fungus Cordyceps chanhua
Source: Front Microbiol. 2024 Jun 27;15:1333793. doi: 10.3389/fmicb.2024.1333793 (PMC11236535; doi:10.3389/fmicb.2024.1333793)

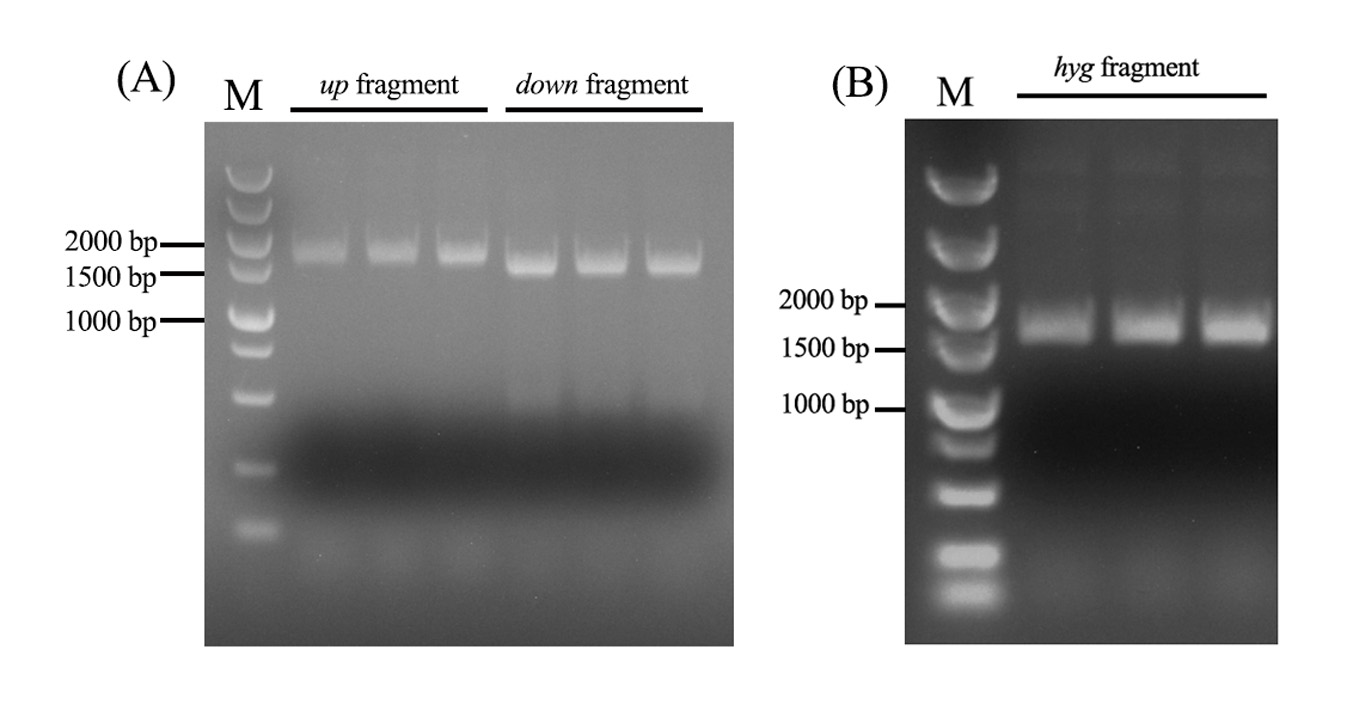

Supplement: SUPPLEMENTARY FIGURE S1 — Construction of deletion cassette by PCR. (A) Up and down fragments of target gene. (B) Hyg fragment (M, Marker). [file Image_1.JPEG]

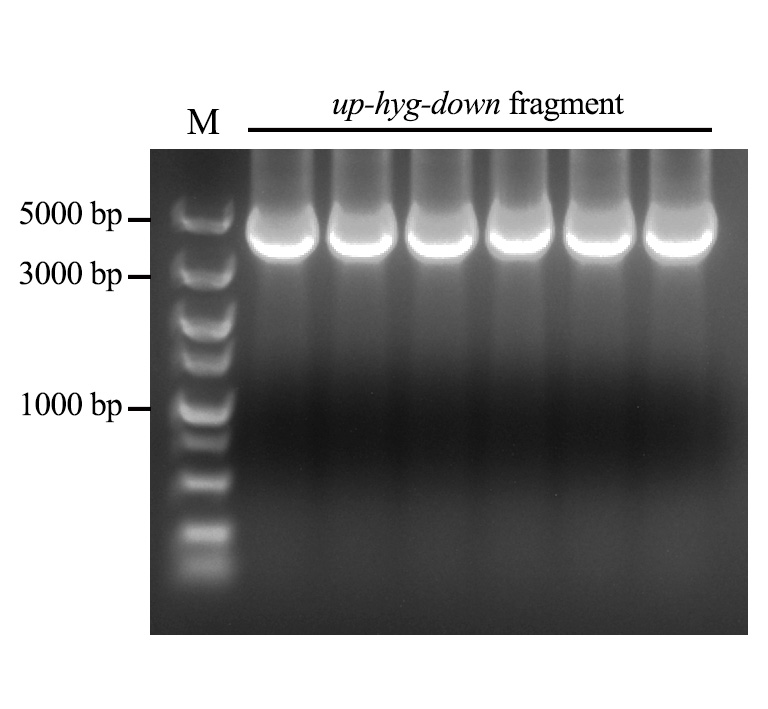

Supplement: SUPPLEMENTARY FIGURE S2 — Up, hyg and down fragments of deletion cassette were assembled by double-joint PCR (M, Marker). [file Image_2.JPEG]
